# Mapping and anatomo-surgical techniques for SMA-cingulum-corpus callosum gliomas; how I do it

**DOI:** 10.1007/s00701-021-04774-7

**Published:** 2021-03-29

**Authors:** Dimitris Klitsinikos, Justyna O. Ekert, Annefloor Carels, George Samandouras

**Affiliations:** 1 Victor Horsley Department of Neurosurgery, The National Hospital for Neurology and Neurosurgery, Queen Square, London, UK; 2 Wellcome Centre for Human Neuroimaging, University College London, 12 Queen Square, London, UK; 3 UCL Queen Square Institute of Neurology, University College London, Queen Square, London, UK

**Keywords:** Brain tumour, Glioma, Corpus callosum, Awake brain mapping, Supplementary motor area

## Abstract

**Background:**

Awake brain mapping paradigms are variable, particularly in SMA, and not personalised to each patient. In addition, subpial resections do not offer full protection to vascular injury, as the pia can be easily violated.

**Methods:**

Mapping paradigms developed by a multidisciplinary brain mapping team. During resection, a combined subpial/interhemispheric approach allowed early identification and arterial skeletonization. Precise anatomo-surgical dissection of the affected cingulum and corpus callosum was achieved.

**Conclusions:**

In SMA-cingulum-CC tumours, a combined subpial/interhemispheric approach reduces risk of vascular injury allowing precise anatomo-surgical dissections. Knowledge of cognitive functions of affected parcels is likely to offer best outcomes.

**Supplementary Information:**

The online version contains supplementary material available at 10.1007/s00701-021-04774-7.

## Relevant surgical/functional anatomy

**Fig. 1:**
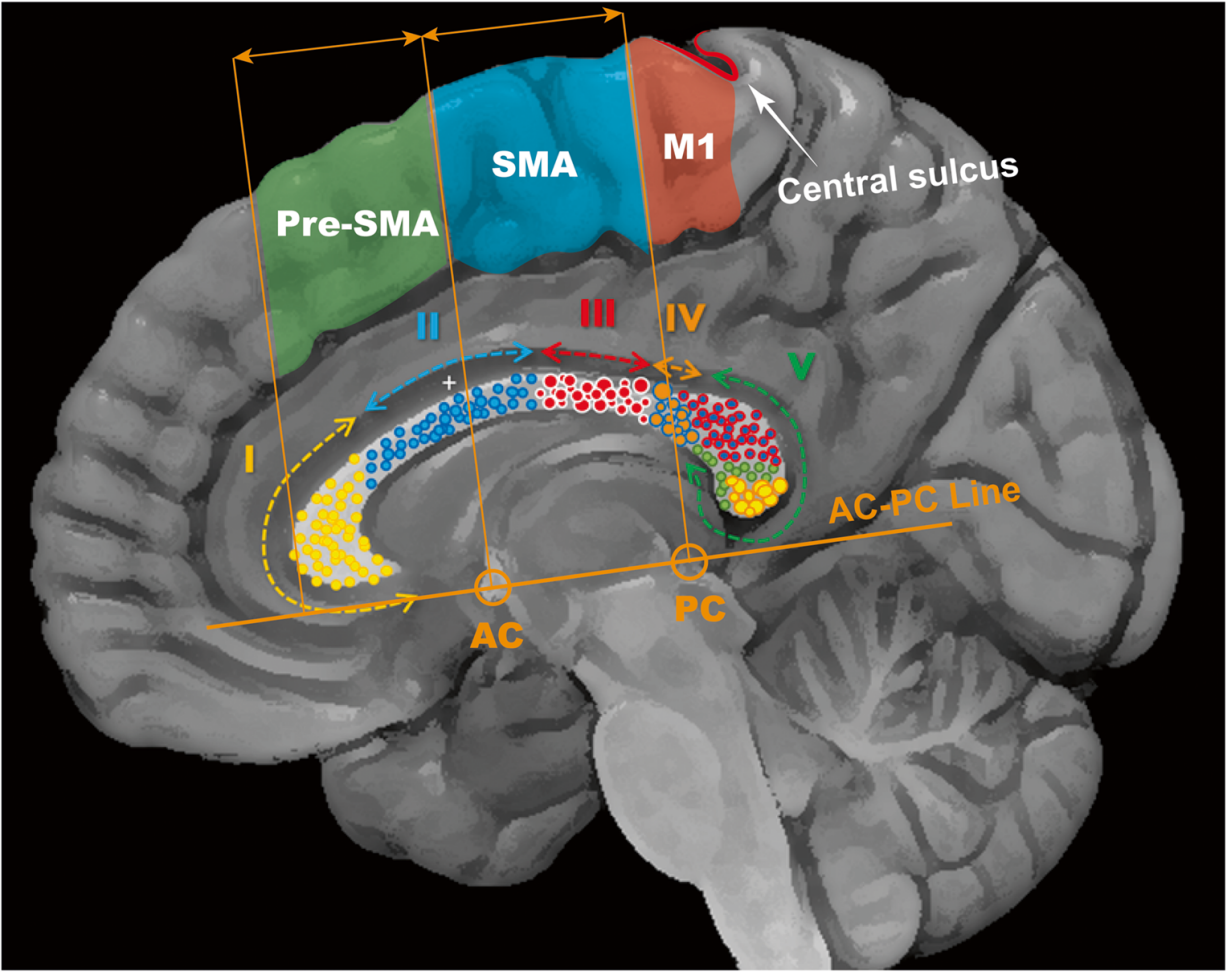
Midsagittal brain view showing critical interhemispheric cortical anatomy. AC, anterior commissure; PC, posterior commissure; M1, primary motor cortex. The somatotopic segmentation model of the CC is based on Hofer [2] I, prefrontal; II, pre-motor; III, motor; IV, sensory; V, temporal, parietal and occipital fibres

The SMA introduced by Penfield 70 years ago as secondary but critical component in voluntary movement following its integration to the medial pre-motor system including the pre-SMA anteriorly and the cingulate gyrus inferiorly [4]. The current pre-SMA and SMA-proper divisions are based on cytoarchitectonic and functional methodologies; interestingly, the border between the two areas coincides with the AC-PC line passing through the anterior commissure (AC), vertically at a line connecting the AC and posterior commissure (PC) (Fig. 1). In addition to motor preparation, monitoring task difficulty and speech articulation, SMA is involved in speech initiation [1]. The corpus callosum (CC) contains somatotopically organised interhemispheric connectivity fibre tracts (Fig. 1) [2]. Currently, there are no validated mapping paradigms for the cingulum or the CC, although developing surgical corridors to the CC requires identifying non-eloquent brain tissue.

### Specific perioperative considerations

**Fig. 2:**
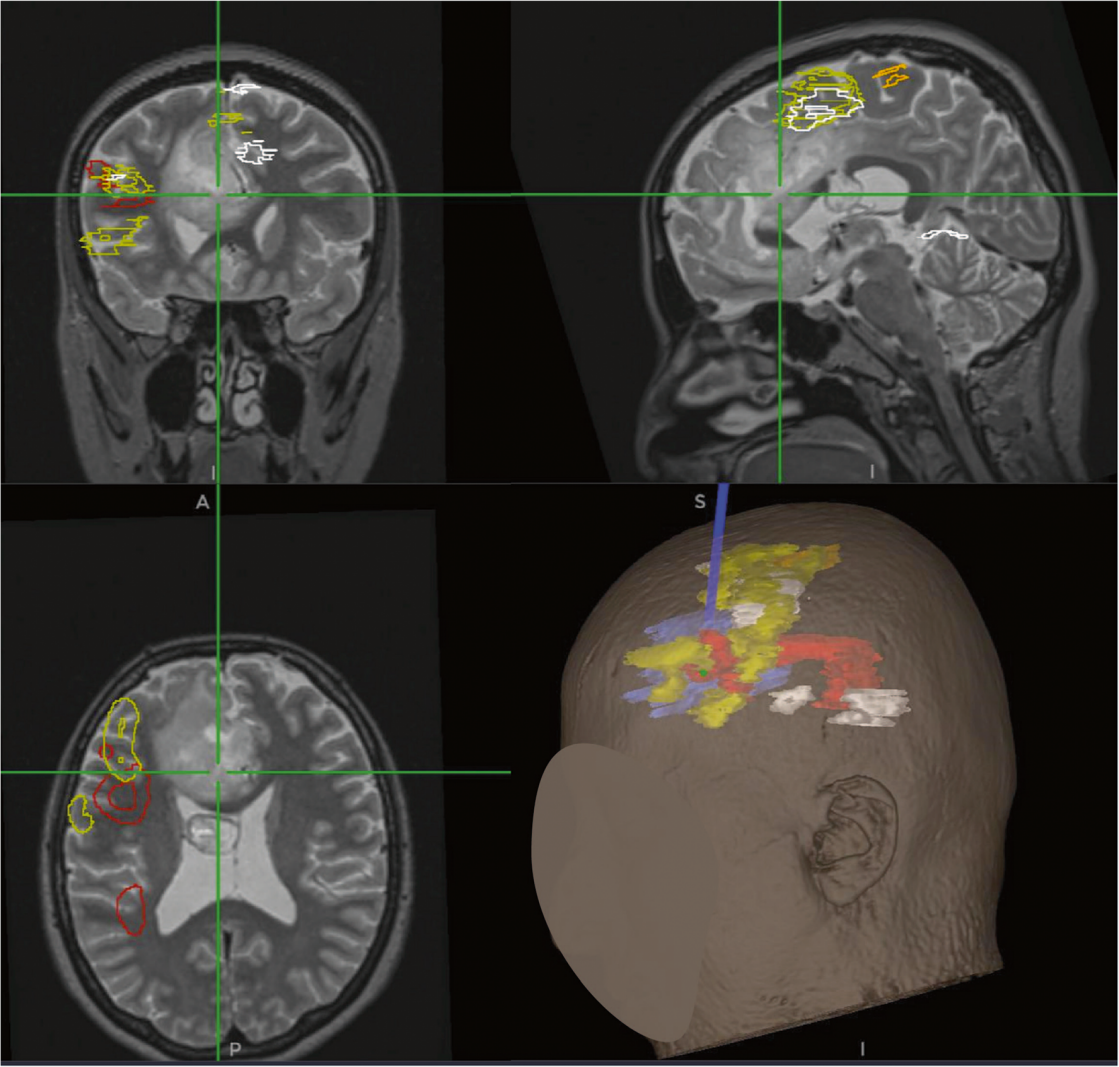
Screenshot demonstrating a non-enhancing glial tumour involving medial pre-SMA, SMA proper, cingulum and genu and body of the CC. fMRI and DTI overlays are outlined manually on axial sequences while the software calculates the 3D volumes (lower right image). Cyan, tumour outline; arcuate fasciculus, red; verbal fluency, yellow; verb generation, white; primary motor cortex, orange

The brain mapping (BM) paradigms of the case were discussed in a novel multidisciplinary (MDT) meeting employed in the senior author’s practice in the last 3 years, with participation of neuropsychologists, speech and language therapists, neuroradiologists, cognitive neuroscientists and neurologists, with analysis of functional imaging and pre-operative assessment data resulting on specific mapping paradigms applicable to the particular patient. Eloquent cortical parcels (verbal fluency, verb generation and primary motor cortex) and subcortical segments (arcuate fasciculus) were encasing the tumour (Fig. 2). The BM-MDT recommended testing the SMA with speech initiation through sentence completion task. Functional MRI (fMRI) and diffusion tensor imaging (DTI) overlays were obtained and loaded into the neuronavigation system (StealthStation S8, Medtronic, Minneapolis, USA) and fused with T2W MRI volumetric sequences. The senior author rendered the overlays to colour-coded volumes, allowing easy intraoperative 3D guidance (Fig. 2, bottom left).

### Description of the technique

#### Anaesthetic technique

Our awake-throughout craniotomy technique has been described [3]. The patient received a small amount of target-controlled infusion (TCI) of propofol and remifentanil targeting blood concentrations of 0.8–1.2 mg/ml and 1–2 ng/ml, respectively [3]. Following Mayfield clamp application, all TCI was stopped. The patient was placed supine on a padded operating table, with the neck slightly flexed and the head in neutral midline position, allowing comfort and tolerance of a potentially lengthy operations. The patient was extensively coached before and throughout surgery on the importance of their active participation and effort.

### Direct electrical stimulation and mapping technique

**Fig. 3:**
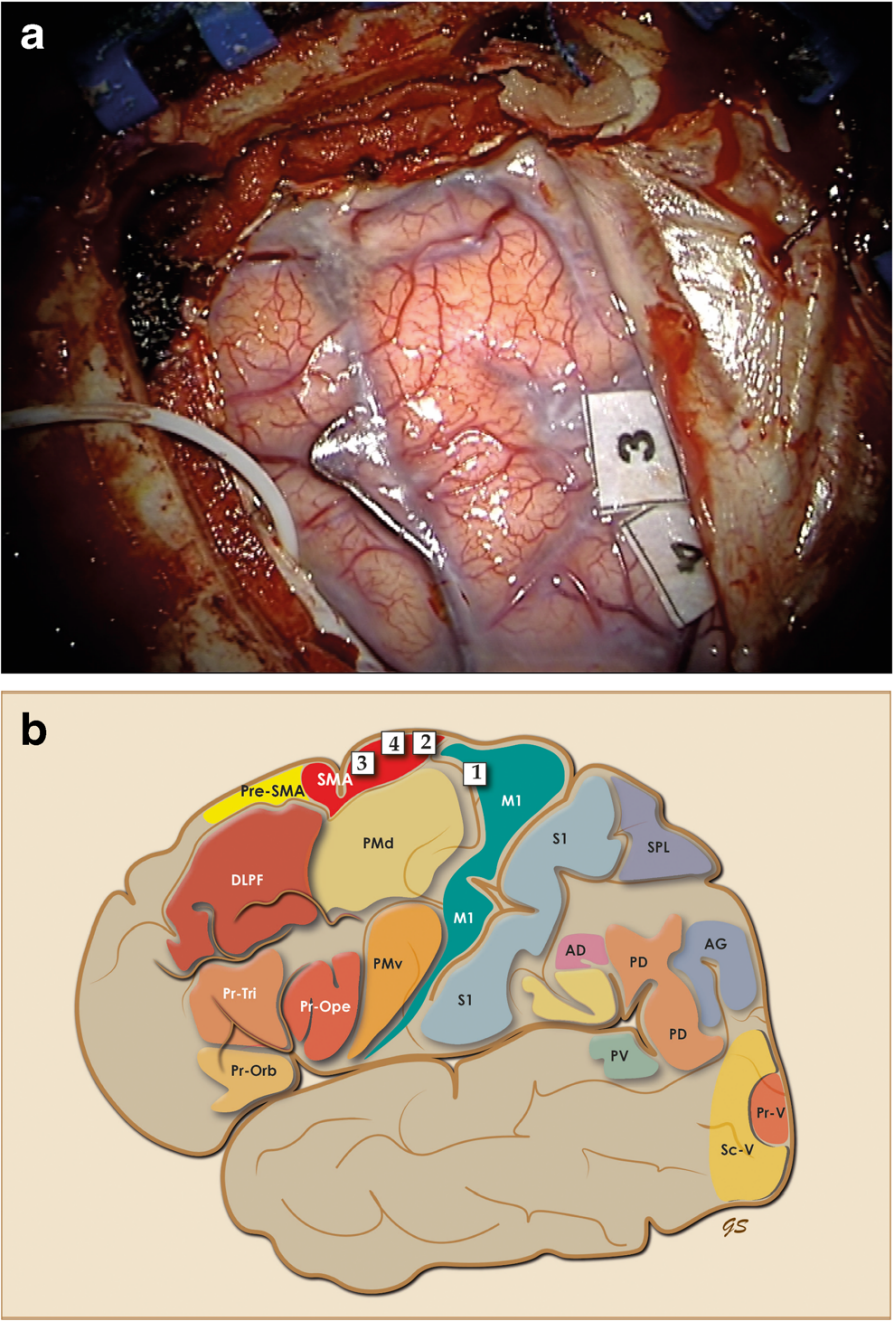
**a** Consistently positive stimulation sites for speech initiation tested with sentence completion test, while no dysarthria, speech arrest or picture naming difficulties were recorded and marked with the sterile tickets 3 and 4. The white cable of the strip electrode is identified in the lower, left corner. **b** Personalized brain map of the patient with the 4 positive cortical stimulation sites, functional outputs and stimulation parameters. #1 M1, movement of elbow, monopolar stimulation at 5 mAmps; #2 SMA, twitching of foot, monopolar stimulation at 5 mAmps; #3 SMA, inability to complete sentences, bipolar stimulation at 5 mAmps; #4 SMA, inability to complete sentences, bipolar stimulation at 5 mAmps. The personalised map template is used in all cases to plan mapping and record mapping findings

Following dural opening under the microscope (Zeiss Kinevo 900, Zeiss, Oberkochen, Germany), a 6-contact strip electrode (Ad-Tech Medical Instrument Corporation, Racine, USA) was inserted for electrocorticography (ECoG) and detection of after-discharge potentials. For motor mapping and language mapping, monopolar stimulation and bipolar stimulation were respectively used (C2 Xtend, inomed, Emmendingen, Germany). The senior author employs a decreasing current stimulation protocol for high frequency, motor testing starting at 10 mAmps while an increasing low-frequency, language testing is used, starting at 2 mAmps with upper limit 5 mAmps or appearance of after-discharge potentials. The M1 was easily identified at 5 mAmps while motor responses were obtained from the posterior SMA with movement slowness and foot twitching. Two discreet areas on SMA, marked with the tickets #3 and #4, produced consistent inability to complete sentences while stimulation of the same area did not affect counting, articulation or picture naming (Fig. 3a and b). All paradigms were proposed by the BM-MDT in the pre-operative group meeting and proven to be accurate.

#### Tumour resection

Once a silent cortical area was identified, following a small corticotomy, the tumour was entered and debulked initially with suction, set at 20 KPa, and the ultrasonic aspiration (CUSA® Clarity, Integra, Plainsboro, USA). The senior author applies the cavitation effect in white matter distally to vessels, while adjacent to pial surfaces, the aspiration effect only is used, as it is safer and finely scalable compared with surgical suction.

**Fig. 4:**
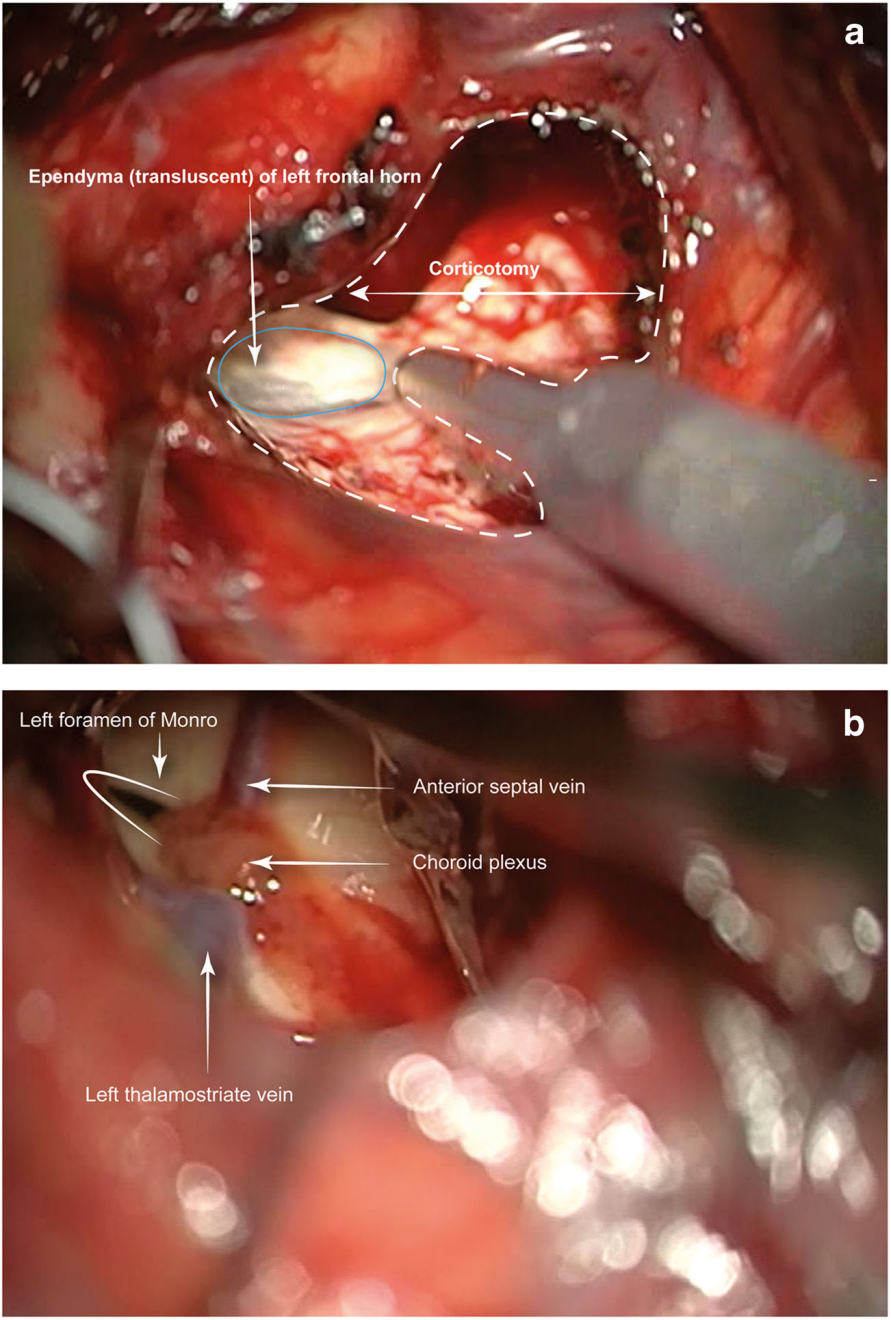
**a** Using the ultrasonic aspirator, on suction only without cavitation, the ependyma can be exposed without entering the ventricle, if necessary. A virtual plane extended to the falx, can help localising the CC. **b** The intraventricular component of the tumour necessitated ventricular entry in this case, with demonstration of key landmarks

For tumours involving the cingulum/CC, neuronavigation is usually of limited assistance while, in our experience, two anatomical landmarks are significantly more accurate: the falx medially and the frontal horn ependyma laterally. The frontal horn does not necessarily need to be de-roofed although intraventricular tumour component removal, in this case, required eventually ventricular entry (Figs. 4a and b). Once both exposed, virtual axial and sagittal orthogonal planes are visualised, intersecting at the CC.

**Fig. 5:**
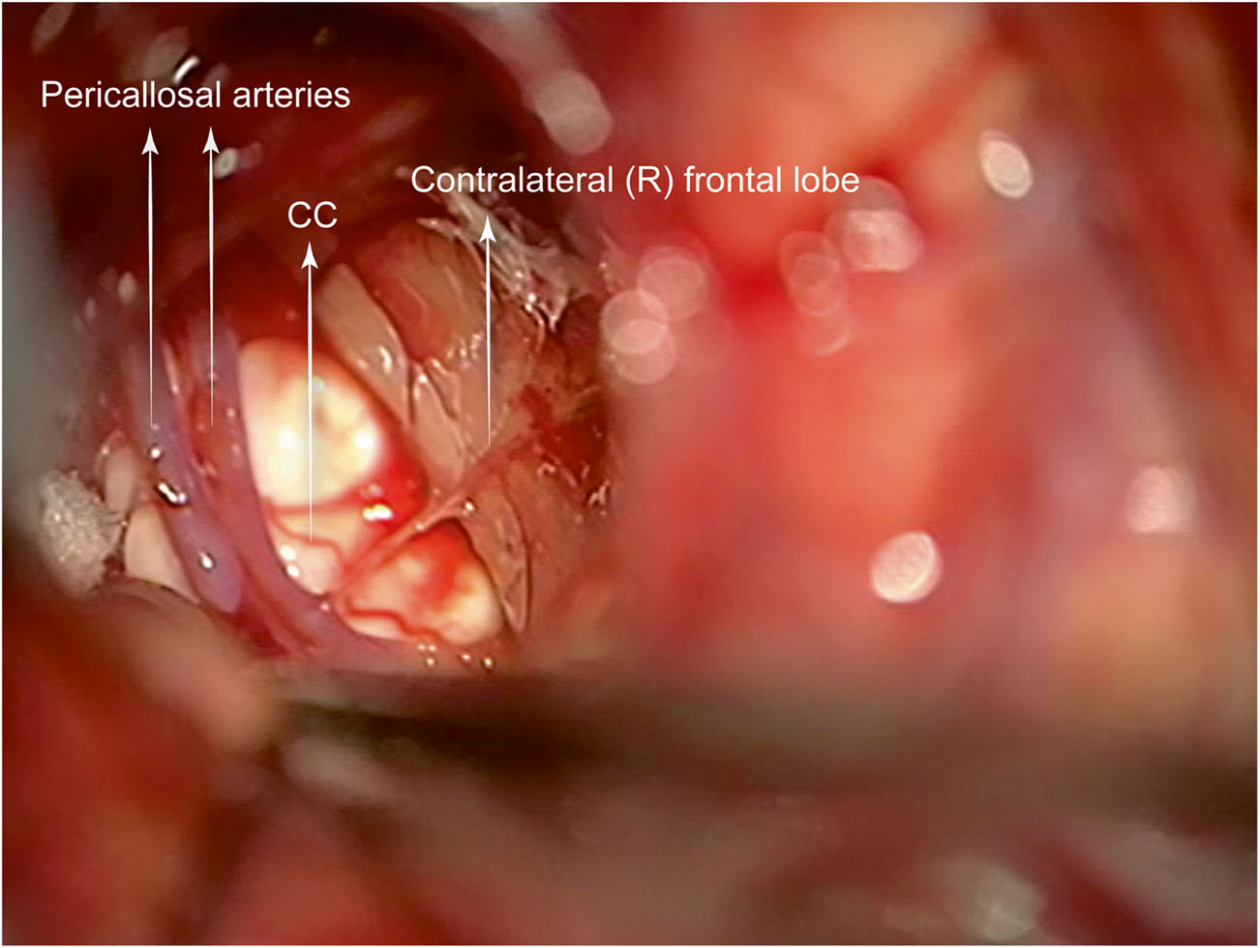
The ipsilateral cingulum was displaced laterally allowing an interhemispheric exposure of the whiter-looking CC and the paired pericallosal arteries

**Fig. 6:**
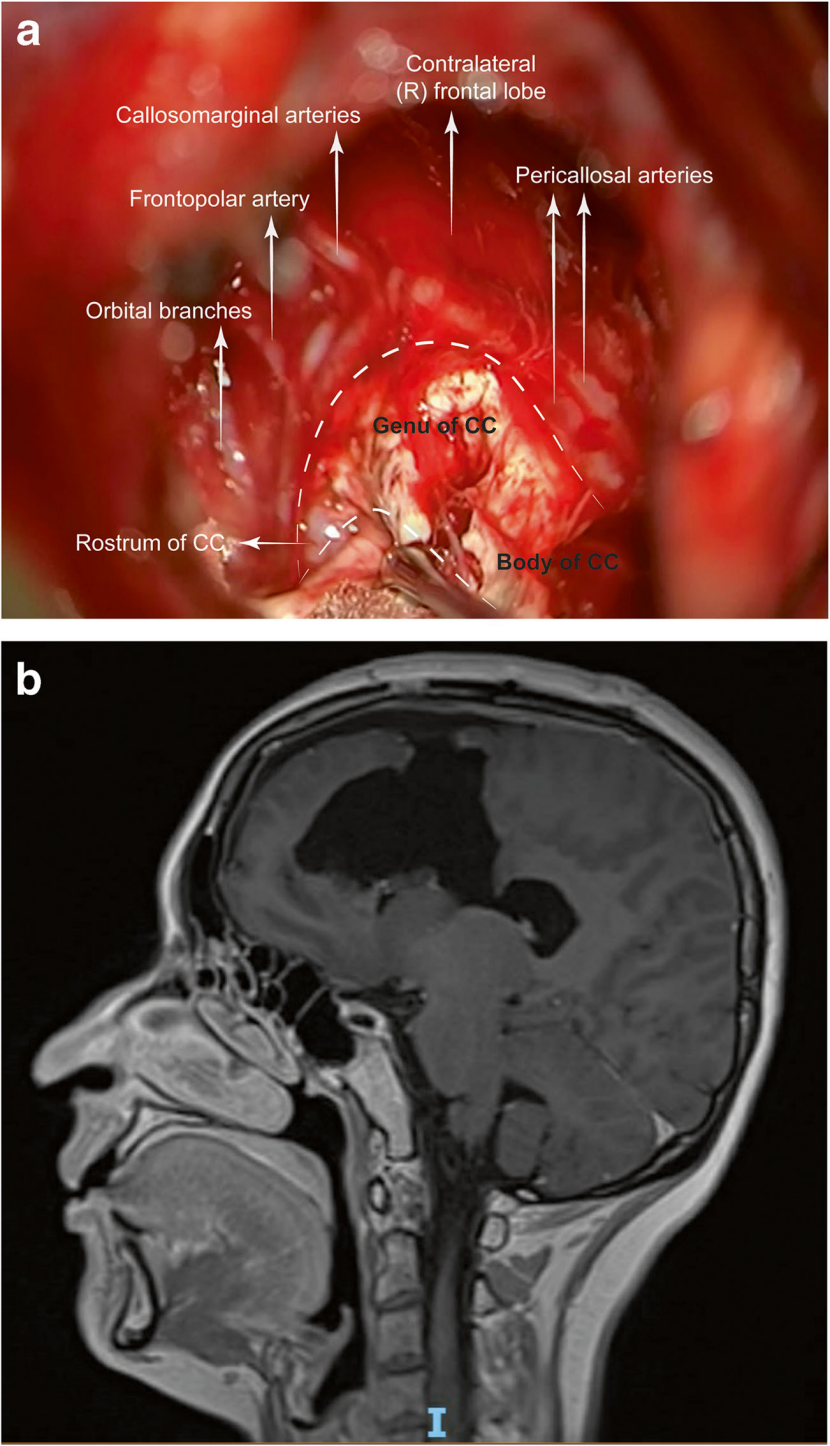
**a** The successful mapping and the combined subpial/interhemispheric dissection allowed clear anatomical visualization of critical structures and safe anatomo-surgical resection of the affected parts of the CC. **b** Sagittal post-operative MRI scan showing resection of the affected regions of the pre-SMA/SMA/cingulum and CC

Most of the tumour removed subpially with the ultrasonic aspirator, without cavitation effect when approaching the midline. A Rhoton #8 microdissector (Integra, Plainsboro, USA) used to dissect the cingulum from the pericallosal arteries. However, early arterial visualisation is critical, as the delicate pia cannot always protect from vascular injury. A partial interhemispheric approach was employed, and the distal anterior cerebral artery (ACA) and the CC clearly visualised (Fig. 5), thereby allowing safe and expedient subpial resection. At the free edge of the falx, the cingulum gently displaced laterally exposing the paired pericallosal arteries against a whiter-looking CC. A careful, anatomo-surgical dissection resulted in clear visualisation of all A2 and A3 branches and the anterior half of the CC (Fig. 6a). Extra care was taken to avoid manipulation-induced vasospasm.

According to anatomical regions, subcortical, continuous monopolar stimulation with mapping suction probe, for corticospinal tract mapping, applied to posteroinferior edges, while bipolar stimulation for language mapping to inferolateral edges, was intermittently performed, with paradigms devised pre-operatively by the brain mapping MDT (Figs. 2, 3b, 6b).

At the end of resection, a comprehensive motor and language testing employing the Boston naming test, sentence completion, verb generation and pyramids and palm trees showed no deviation from the pre-operative baseline.

### Indications

Tumours affecting SMA, cingulum and CC

### Limitations

Developing specific mapping paradigms per brain region requires experienced, MDT teams. In addition, patients must be motivated and cooperating throughout the operation, to allow successful initial and subsequent mapping.

### How to avoid complications

Two types of complications can be observed. (1) Vascular injury to the superior sagittal sinus (SSS) or distal ACA. SSS injury risk is minimised if the craniotomy of performed 1 cm laterally, without exposing the SSS. The risk of ACA injury is minimised with a combined subpial/inter-hemispheric technique allowing early vessel identification and safer, expedient subpial resections.

(2) Injury of eloquent functions from incomplete or inaccurate intraoperative testing. In particular, extensive variability is documented in selection of intraoperative tests for language and other cognitive functions [5]. A large-scale specialists survey conducted by our group, on selection and interpretation of intraoperative tests, documented lack of agreement in all domains regardless of specialism or years of experience [5]. Our personalised model of mapping within the context of a BM-MDT conferred personalised testing paradigms.

### Information given to the patient

The patient is given specific written information, applicable to the senior author’s practice, with description of each step of the ABM, including positioning, drilling, mapping and expectation setting. The patient is coached and supported before and during ABM.

## 10 key point summary

- Awake-throughout craniotomy avoids intubation/extubation cycles and allows smooth phase transitions and enhances patient comfort.
- Patient coaching before and during surgery is critical for successful ABM.
- Each case is studied individually by an MDT team, and mapping paradigms are personalised based on pre-operative neuropsychological and functional neuroimaging testing.
- Volume rendering of functional overlays facilitates neuronavigation.
- The dominant SMA is involved in movement and speech initiation and can be tested consistently.
- Monopolar and bipolar stimulations are best for motor and language testing, respectively.
- Resection of targeted diseased segments of the CC, in the zones I, II, or III are unlikely to result in deficits.
- The distal cerebral arteries are relatively small and can be easily damaged resulting in stroke.
- Subpial resection is routine but interhemispheric vessel inspection enhances safety and vessel protection.
- Anatomo-surgical dissections are likely to offer best surgical orientation and reduced complications.

## Supplementary Information

ESM 1 Key surgical steps and anatomo-surgical dissection of the dominant SMA/cingulum/CC glioma (MP4 265744 kb)
